# Growth performance of Indonesian three-breed cross chicken associated with growth hormone and insulin-like growth factor 2 genes

**DOI:** 10.14202/vetworld.2023.2471-2478

**Published:** 2023-12-20

**Authors:** Harini Nurcahya Mariandayani, Sri Darwati, Isyana Khaerunnisa, Vivitri Dewi Prasasty

**Affiliations:** 1 Department of Biology, Faculty of Biology and Agriculture, Universitas Nasional, Jakarta 12520, Indonesia; 2 Department of Animal Production and Technology, Faculty of Animal Husbandry, Bogor Agricultural University, Bogor, West Java 16680, Indonesia; 3 Research Center for Applied Zoology, National Research and Innovation Agency, Bogor, West Java 16912, Indonesia; 4 Department of Pathobiological Sciences, School of Veterinary Medicine, Louisiana State University, Baton Rouge, Louisiana 70803, USA

**Keywords:** body weight, domestic chicken crossbreed, feed conversion, growth hormone gene, insulin-like growth factor 2 gene

## Abstract

**Background and Aim:**

Poultry, such as chickens, is an important source of animal protein, producing eggs and meat. Local chickens are able to adapt to the hot weather and become more resistant to disease. However, it has relatively slow growth and low egg production. These problems can be overcome through holding selection and crossing. Local chicken productivity is slow and low based on chicken growth. There is a need to examine the factors that influence growth and productivity. Therefore, this study aimed to evaluate crossbreed chicken growth performance, including body weight (BW), BW gain, feed intake, and feed conversion.

**Materials and Methods:**

DNA was extracted from 40 chickens with the growth hormone (GH) gene and 40 chickens with the insulin-like growth factor 2 (IGF2) gene, followed by a polymerase chain reaction. Genotyping was performed using restriction fragment length polymorphism analysis. In animal selection and phenotypic data collection, 80 chickens from Sentul, Kampung, and Kedu were used to produce the second-generation three-crossbreed chickens (F2) using the GH gene.

**Results:**

Growth hormone is a very relevant gene in chicken performance traits. Growth hormone and IGF2 genes regulate chicken production. This study presents the second-generation growth features of three-crossbreed chickens derived from Sentul, Kampung, and Kedu, all of which are native to Indonesia (F2). A statistically significant (p = 0.05) improvement in BW, weight gain, feed intake, and feed conversion over a 12-week period was observed when the animals were allowed free access to regular feed. Analysis of variance results indicated a significant (p = 0.0001) interaction between the 12-week period and GH and IGF2 gene sensitivities of different chicken breeds.

**Conclusion:**

Crossbreed chicken growth performance increased within 12 weeks. This study highlighted the need to improve the productivity and breeding of domestic crossbred chickens to contribute to the Indonesian conservation and genetic diversity program.

## Introduction

Local Indonesian chicken is a commodity mainly maintained by the community, especially in rural areas [1]. Therefore, local chickens play a significant role for the people of Indonesia because they can meet economic needs and increase farmers’ income and animal protein sources [2]. Another unique advantage of local chickens is their ability to survive in hot environment with low feed quality and develop disease resistance [3]. Therefore, local chickens can grow rapidly with the advantages already in place [3, 4]. However, domestic chicken productivity is generally low due to slow growth, low spawning, and extensive care systems [5, 6]. The growth of local chickens can be increased through selection and cross-breeding [7]. Domestic chicken crossings are expected to increase productivity through cross-group crossings, thereby achieving high levels of heterogeneity, which may further improve genetic quality [8].

Local Indonesian chicken varieties, such as Sentul, Kampung, and Kedu [9–11], play a pivotal role in the country’s poultry industry. This study delves into the complex factors influencing the growth and productivity of these indigenous chicken breeds, particularly those with slow growth and low output [12, 13]. The significance of this study lies in its potential to provide valuable insights for improving poultry farming practices and fostering increased food production and security. A meticulous examination of genetic intricacies linked to growth patterns in domestic chickens resulting from cross-breeding efforts. Moreover, hormonal influences, in particular the growth hormone (GH) gene is recognized as a pivotal candidate in livestock development [14, 15]. The GH gene’s influence on essential somatic cells, including muscle cells, bone cells, epithelial cells, and fibroblasts, underscores its significance in shaping growth trajectories [16, 17]. Additionally, the exploration of the interplay between insulin-like growth factor 2 (IGF2) and GH genes shed light on their profound effect on the growth and overall quality of poultry meat. The importance of selection and cross-breeding as desirable methods for enhancing poultry growth performance. The expectation is that these methods will also increase productivity and achieve a high level of heterogeneity, ultimately elevating genetic quality. The strategic crossing of domestic and exotic chicken breeds is particularly emphasized as a means to improve growth performance in domestic chickens regulated by the GH and IGF2 genes [18, 19].

This study aimed to investigate the second-generation (F2) growth performance of Sentul, Kampung, and Kedu chickens using a cross. The measured productivity parameters were BW, daily weight gain, feed consumption, and feed conversion from 0 to 12 weeks. This study is expected to provide a solution for Indonesian farmers by providing domestic chickens that are superior to other domestic chickens. By understanding and leveraging these genetic factors, this study could contribute to the development of effective strategies for advancing poultry farming, promoting sustainable food production, and enhancing overall food security.

## Materials and Methods

### Ethical approval

The study was approved by the Animal Ethics Committee at Bogor Agricultural University (approval no. 22-2016IPB) for the experimental procedures involving animals.

### Study period and location

This study was conducted from January 2021 to December 2022 in Department of Animal Production and Technology, Faculty of Animal Husbandry, Bogor Agricultural University, Bogor, West Java, Indonesia.

### DNA extraction and polymerase chain reaction (PCR)

DNA extraction [2] was performed on 80 chickens, comprising 40 from GH and 40 from IGF2. Primers were used in accordance with the information given in Table-1. Polymerase chain reaction involves several steps. First, predenaturation was performed at 95°C for 5 min. This was followed by denaturation for 35 cycles at 95°C for 10 s. Amplification was performed at 60°C for 20 s. This was followed by an extension step at 72°C for 30 s and a final extension at 72°C for 5 min. The PCR experiment was conducted using an Eppendorf^®^ AG 22331 thermocycler (Hamburg, Germany). The PCR mixture consisted of 0.3-μL primers, 6.2-μL distilled water (DW), and 7.3-μL GoTaq^®^ Green Master Mix (Promega, Madison, US). The PCR products were subjected to restriction enzyme digestion. The EcoRV enzyme was used to digest GH, whereas IGF2 was digested at 37°C using *Nla*III. The restriction mix consisted of 5-μL PCR products, 0.3-μL restriction enzymes, 0.7-μL 1 buffer, and 1-μL DW. The PCR-restriction fragment length polymorphism (PCR-RFLP) results were analyzed using an electrophoresis station and visualized using an AlphaImager^®^ EP ultraviolet (UV) transilluminator (Santa Clara, US). Table-1 lists the primer sequences that were designed.

**Table-1:** Primer sequences for GH and IGF2 genes.

Target gene	Primer sequence
GH	f: 5’- ATGTCTCCACAGGAACGCAC -3’
	r: 5’- GCTCTGTAAGCTGAGCACCAC -3’
IGF2	f: 5’- GCTGGGGACCCAATAG AACC -3’
	r: 5’- TCCCCAGGAGATCACAAATCG -3’

f=Forward, r=Reverse, GH: Growth hormone, IGF=Insulin-like growth factor 2

Genotyping was performed using RFLP. Four μL of the amplicon was added to the mixture, along with 0.9-μL DW, 0.7-μL buffer, and 0.4-μL restriction enzymes. The GH intron gene was cleaved into three regions using *Eco*RV. The cleavage reaction was then incubated at 37°C for 4 h. The DNA sample was electrophoretically run on a 2% agarose gel at 100 V for 35–45 min at a volume of 5 μL. Subsequently, electrophoretic DNA samples were observed under UV light. We compared the DNA fragments observed in the electrophoretic results with the fragments observed in the marker.

### Animal selection and phenotypic data collection

Eighty chickens were used as three-crossbreed chickens (F2, a second-generation) from indigenous chickens of Sentul, Kampung, and Kedu. These 80 chickens were divided into two primary groups based on two significant genes, 40 chickens from the GH gene with genotypes of AG (n = 14) and GG (n = 26). The other 40 chickens were from the IGF 2 group with TC (n = 25), CC (n = 7), and TT (n = 8) genotypes.

Hatched eggs were collected every morning and evening from Sentul, Kampung, and Kedu crossed chickens (F2). The eggs were hatched every week in the hatching machine. Day-old chickens (DOC) were maintained in a cage equipped with lights, feed, and drinking water.

Feeds were given twice daily in the morning and evening. Drinking water was provided *ad libitum* during maintenance. Commercial feed in the form of crumble was given to DOC for 3 weeks during the chicken starter phase. Four-week-old chickens were given a mixture of commercial feed and bran at a commercial feed: Bran ratio of 80:20. Chickens aged 5–12 weeks were fed a combination of commercial feed and bran with a commercial feed: Bran ratio of 60:40.

### Statistical analysis

The weekly data record included BW growth, daily body growth, feed intake, and feed conversion. Correlation, regression, one-way analysis of variance (ANOVA), and independent sample t-test were performed using GraphPad Prism 8 software (https://www.graphpad.com/). An independent sample t-test was used to compare the average BW, body growth, feed intake, and feed conversion among the six chicken populations [20]. Correlation and relationship analyses were conducted among the five chicken populations based on phenotypic parameters. The visual observation scoring method was analyzed based on BW, daily BW gain, feed intake, and feed conversion during 0–12 weeks to determine the significant difference (p = 0.05).

## Results

### Polymorphisms of the GH and IGF2 genes

**Figure-1:**
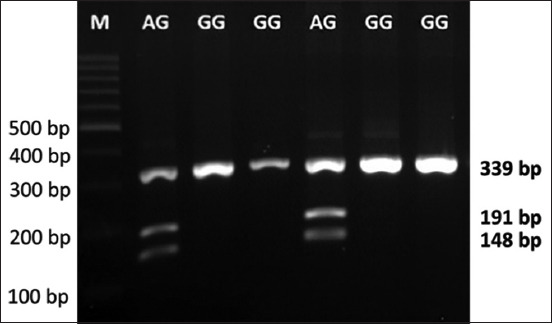
Electropherogram of domestic crossbreed chicken using polymerase chain reaction-restriction fragment length polymerase chain reaction growth hormone|*Eco*RV.

Partial fragments of GH in 40 chickens were successfully amplified using PCR-RFLP and showed three bands: 339 kb, 191 kb, and 148 bp, using an electropherogram (Figure-1). The lengths of the PCR products with reference sequences were deposited in GenBank (GenBank accession number: AY461843.1). Two genotypes (AG and GG) were identified as genotypic profiles.

**Figure-2:**
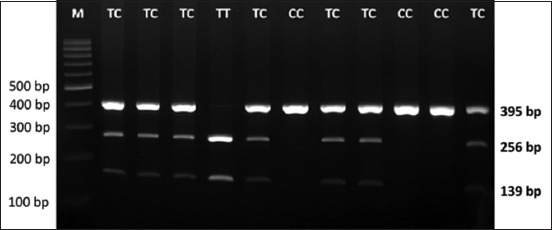
Electropherogram of domestic crossbreed chicken using polymerase chain reaction-restriction fragment length polymerase chain reaction insulin-like growth factor|*Nla*III.

Partial fragments of IGF2 in 40 chickens were also effectively amplified using an electropherogram and showed three bands, 395 bp, 256 kb, and 139 bp (Figure-2). Three genotypes (CC, TC, and TT) were identified as genotypes.

###  Influence of gene polymorphism on growth traits

**Figure-3:**
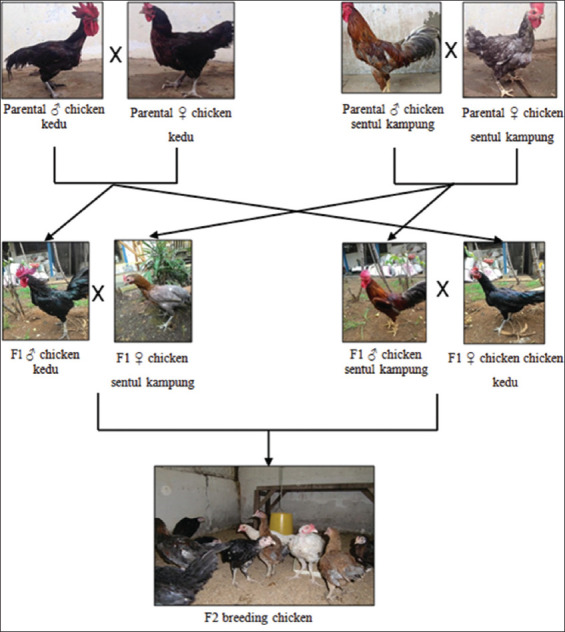
Breeding line of parental, F1 and F2 chicken kedu (KeduSK) and chicken sentul kampung (SKKedu).

A schematic breeding line of parental, F1, and F2 chickens between KeduSK and SKKedu can be represented as the parental generation involved in purebred KeduSK and SKKedu chickens. F1 generations were obtained by crossing KeduSK roosters with SKKedu roosters. As shown in Figure-3, the F2 generation was obtained by crossing F1 roosters with F1 hens.

**Figure-4:**
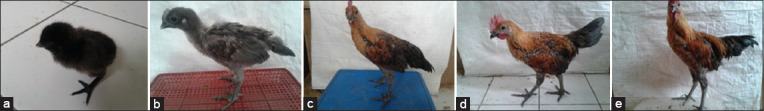
Growth performance of F2 breed cross chicken from (a) day-old chicken; (b) 2 weeks old; (c) 4 weeks old; (d) 8 weeks old; (e) 12 weeks old.

Figure-4 shows different phenotypes of F2 crossbred chickens at different growth stages.

At the age of 1 day, the chickens have just hatched, and are tiny with a small BW and black feathers. However, they begin to experience rapid growth within the first 2 weeks, with BW increasing much faster within the 75–150 g range. At 4 weeks of age, the chickens weighed within the range of 230–270 g, and their phenotype was more developed with a distinctive body shape. At 8 weeks of age, the chickens developed significantly and weighed within the 300–700 g range, and their feathers fully developed. At 12 weeks of age, chickens weighed within the range of 450–1200 g, and their phenotype was almost fully developed.

**Figure-5:**
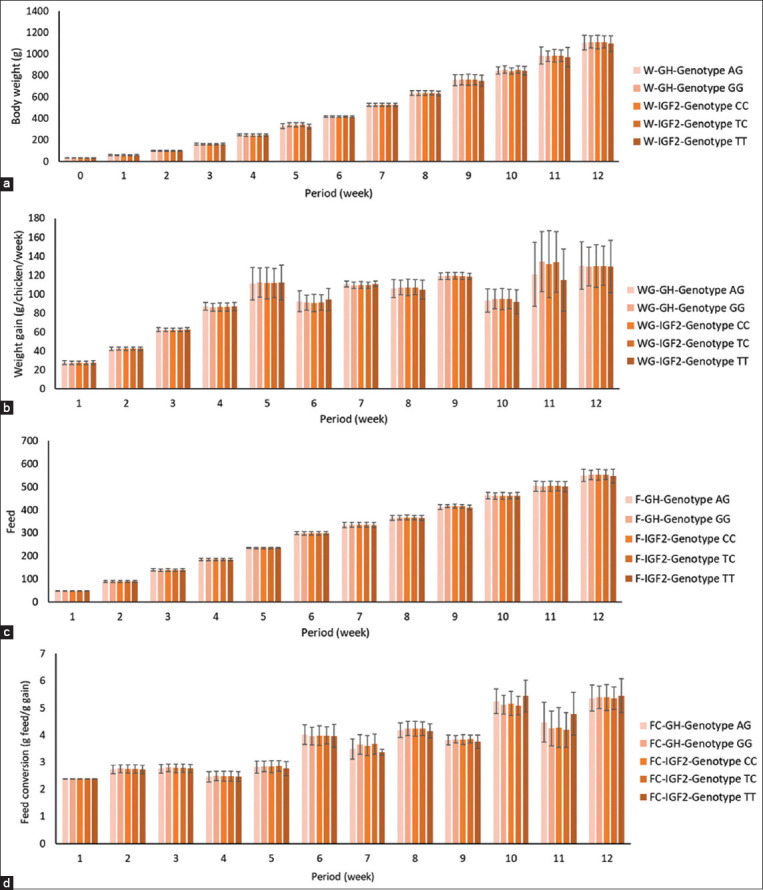
Histogram diagram of chicken population based on (a) average body weight; (b) body growth; (c) feed intake; and (d) feed conversion. W=Average body weight, WG=Body weight gain, F=Feed intake, FC=Feed conversion.

Figure-5 shows the average BW, BW gain, feed intake, and feed conversion of crossbreed chickens. Growth performance increased for all chicken populations with GH and IGF2 levels during 12 weeks. The BW and feed intake performance of the chickens increased during the 12-week period. In addition, BW gain and food conversion performance increased slightly during the 5^th^ week and decreased slightly during the 10^th^ week for GH, whereas IGF2 increased slightly during the 10^th^ week.

**Figure-6:**
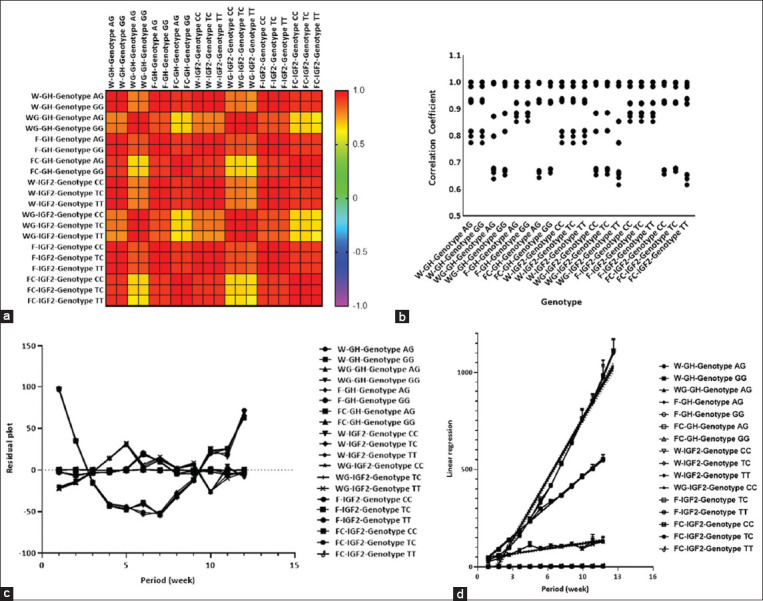
The statistical analysis expressed the correlation and relationship between the chicken population versus the time within 12 weeks. (a) Pearson’s correlation in heatmap; (b) grouped superimposed-scatter plot; (c) residual plot versus a time within 12 weeks; (d) Simple relationship under linear regression of chicken population versus period within 12 weeks. W=Average body weight, WG=Body weight gain, F=Feed intake, FC=Feed conversion.

Pearson’s correlation analysis revealed a positive correlation (Figure-6) between the groups.

However, the groups also had a positive correlation among other populations, as shown in the heatmap (Figure-6a). Some populations have also demonstrated a significant correlation between genes and genotypes, whereas others show weak correlations. Correlations within the internal population also showed a homogenous distribution in each group, indicating that all chicken populations grew with slight variation (Figure-6b). Moreover, analysis of residual plots in terms of autocorrelation and scatter plots of residuals versus time revealed wide deviations for some groups, whereas none for others (Figure-6c). Population growth rates were calculated as the slope of the linear regression of natural logarithms of chicken versus period length when the populations grew at their maximal rates (Figure-6d).

One-way ANOVA of BW, weight gain, feed intake, and feed conversion of crossbreed chickens differed significantly (p < 0.05) weekly in each chicken group. The average BW, weight gain, feed intake, and conversion at various time points were calculated using weekly averages. These parameters significantly increased (p < 0.0001) with the age of the chickens, with slight differences among GH and IGF2 genotypes over time, as shown in Table-2.

**Table-2:** Genotype characteristics of GH and IGF2 genes from three-breed cross chicken.

No.	Description	Gene type	p-value	t	df	R^2^
1	W-GH-Genotype AG	GH	<0.0001	52.51	19	0.977
2	W-GH-Genotype GG		<0.0001	52.29	19	0.978
3	WG-GH-Genotype AG		<0.0003	30.22	19	0.751
4	WG-GH-Genotype GG		<0.0002	30.77	19	0.774
5	F-GH-Genotype AG		<0.0001	81.93	19	0.998
6	F-GH-Genotype GG		<0.0001	82.41	19	0.998
7	FC-GH-Genotype AG		<0.0001	29.80	19	0.848
8	FC-GH-Genotype GG		<0.0001	31.11	19	0.844
9	W-IGF2-Genotype CC	IGF2	<0.0001	52.23	19	0.977
10	W-IGF2-Genotype TC		<0.0001	52.26	19	0.977
11	W-IGF2-Genotype TT		<0.0001	52.83	19	0.978
12	WG-IGF2-Genotype CC		<0.0001	30.94	19	0.775
13	WG-IGF2-Genotype TC		<0.0002	31.13	19	0.777
14	WG-IGF2-Genotype TT		<0.0001	28.48	19	0.716
15	F-IGF2-Genotype CC		<0.0005	82.20	19	0.998
16	F-IGF2-Genotype TC		<0.0001	82.24	19	0.998
17	F-IGF2-Genotype TT		<0.0001	82.29	19	0.998
18	FC-IGF2-Genotype CC		<0.0001	30.61	19	0.843
19	FC-IGF2-Genotype TC		<0.0001	31.59	19	0.841
20	FC-IGF2-Genotype TT		<0.0001	28.14	19	0.844

IGF2=Insulin-like growth factor 2, GH=Growth hormone

## Discussion

Native chickens, with over 30 distinctive breeds, are the flagship domestic animal breeds of Indonesia [15, 21]. In addition to having a phenotypic character similar to that of a local chicken, developing this new chicken strain aims to achieve faster growth than local chicken. To increase the production of domestic chicken meat [22, 23], it can be harvested more quickly. Hybrid chicken has a significant growth rate within 12 weeks. In addition, BW gain, feed intake, and feed conversion showed a significant increasing trend compared with the first week. Growth hormone and IGF2 genes are candidate genes for growth performance in chickens [24–26]. Therefore, these genes are usually considered candidate genes in the genetic analysis of complex animal traits such as growth rate, body composition, and fat deposition [27]. This study clearly demonstrated the relationship between GH and IGF2 SNPs and the growth traits of hybrid chickens.

To differentiate chicken breeds, native Indonesian chickens are known as Kampung or native chickens (nonbreed chickens) [28, 29]. However, emphasizing these patterns of muscle or BW evolution with the age of the poultry within each breed, the profiles of weight gain, feed intake, and feed conversion over time did not differ among the five examined genotypes of chicken breeds.

Despite the emergence of advanced genetic engineering technology, the relationship between phenotypes and genotypes remains unclear. As a result, traditional approaches, including selection and breeding techniques, are still indispensable to enhance the genetic attributes of animals [30, 31]. One additional constraint encountered in the advancement of native chicken production in Indonesia is the scarcity of fundamental data on production, reproduction, heritability, genetic correlation, and phenotypic correlation [32, 33]. Hence, the process of crossing native chickens necessitates the assessment of genetic parameters, where the backcross population is a unified breed population [34, 35].

The average values for BW, weight gain, food intake, and food conversion were higher in breed chickens than in local chickens [36, 37]. This study successfully investigated crossbred chickens, yielding valuable and comprehensive baseline data that can be effectively used in selecting and breeding programs to enhance growth traits in indigenous chicken populations [38, 39]. The three crossbred chickens exhibited notably elevated growth features, suggesting the superior growth parameters of the crossbred chicken [4]. A significant effect of genotype and the interplay between genotype and BW in crossbred chickens across various developmental stages have been reported [4]. Hybrid chickens exhibited increased feed intake across all developmental stages and demonstrated a superior feed conversion ratio during weeks 8–12. However, during weeks 1–10, a more favorable feed conversion ratio was observed. As a result, this crossbred chicken can be considered to enhance growth characteristics during the early stages of life.

Similarly, domestic and crossbred chickens are available in West Java, Central Java, and East Java [40]. Native chicken products are well-known brands in Indonesian markets. Thus, local chicken prices in Indonesia are higher than those of broilers [40, 41]. The main obstacles to local chicken development are low carcass proportions due to low growth rates due to low yields, low BW, high mortality risk from Newcastle disease, small body and egg size, low egg yield (ratio), and egg yield (yield), low egg production rates, and high feed cost [42, 43]. However, further studies are needed to explore other factors such as disease resistance and adaptability of crossbred chickens to different environments through intensive selection. The results of these studies and the results presented here could be a significant prelude to improving chicken farms.

## Conclusion

Chicken GH gene is widely recognized as the most influential gene affecting chicken performance traits. This is mainly due to its important role in promoting growth. Growth hormone and IGF2 are genes that affect chicken growth. The growth traits of second-generation (F2) three-breed cross chickens, specifically those obtained from indigenous chicken breeds such as Sentul, Kampung, and Kedu chickens were examined. A significant increase (p < 0.05) in BW, feed intake, and feed conversion was observed during the 12-week study period. It was observed when the subjects were provided with an *ad libitum* diet consisting of standard feed. ANOVA analysis revealed a significant interaction between genotype breed chickens, GH and IGF2 genes, and duration over 12 weeks (p = 0.0001). Therefore, it would be beneficial to conduct a study with a larger sample size of hybrid chickens in the future to further validate the results. Confirmation of research findings by conducting studies with a larger population is necessary.

## Authors’ Contributions

HNM and IK: Conceptualization of the study, interpretation of data, and writing—original draft preparation. HNM, IK, and SD: Methodology and Data analysis. HNM and VDP: Software. HNM, IK, SD, and VDP: Validation, investigation, visualization, and project administration. HNM, IK, VDP, and SD: writing, review, and editing. All authors have read, reviewed, and approved the final manuscript.
